# Results of an open-label phase 1b study of the ERK inhibitor MK-8353 plus the MEK inhibitor selumetinib in patients with advanced or metastatic solid tumors

**DOI:** 10.1007/s10637-022-01326-3

**Published:** 2023-04-11

**Authors:** Anastasios Stathis, Anthony W. Tolcher, Judy S. Wang, Daniel J. Renouf, Lin-Chi Chen, Leah H. Suttner, Tomoko Freshwater, Andrea L. Webber, Tapan Nayak, Lillian L. Siu

**Affiliations:** 1 Oncology Institute of Southern Switzerland, EOC, via A. Gallino 12, Bellinzona 6500, Switzerland; 2 NEXT Oncology, San Antonio, TX USA; 3 Florida Cancer Specialists/Sarah Cannon Research Institute, Sarasota, FL USA; 4 BC Cancer-Vancouver Center, Vancouver, BC Canada; 5 Merck & Co., Inc., Rahway, NJ USA; 6 Princess Margaret Cancer Centre, Toronto, ON Canada

**Keywords:** ERK inhibitor, MEK inhibitor, MK-8353, Selumetinib, Solid tumors, Tolerability

## Abstract

Aim: We evaluated MK-8353 (small molecule inhibitor of extracellular signal-regulated kinase 1/2) plus selumetinib (mitogen-activated extracellular signal-regulated kinase 1/2 inhibitor) in patients with advanced solid tumors. Methods: This phase 1b, open-label, dose-escalation study (NCT03745989) enrolled adults with histologically/cytologically documented, locally advanced/metastatic solid tumors. MK-8353/selumetinib dose combinations were intended to be investigated in sequence: 50/25, 100/50, 150/75, 200/75, 200/100, and 250/100. Each agent was administered orally BID 4 days on/3 days off in repeating cycles every 21 days. Primary objectives were safety and tolerability and to establish preliminary recommended phase 2 doses for combination therapy. Results: Thirty patients were enrolled. Median (range) age was 61.5 (26−78) years and 93% had received previous cancer therapy. Among 28 patients in the dose-limiting toxicities [DLT]-evaluable population, 8 experienced DLTs: 1/11 (9%) in the MK-8353/selumetinib 100/50-mg dose level experienced a grade 3 DLT (urticaria), and 7/14 (50%) in the 150/75-mg dose level experienced grade 2/3 DLTs (n = 2 each of blurred vision, retinal detachment, vomiting; n = 1 each of diarrhea, macular edema, nausea, retinopathy). The DLT rate in the latter dose level exceeded the prespecified target DLT rate (~30%). Twenty-six patients (87%) experienced treatment-related adverse events (grade 3, 30%; no grade 4/5), most commonly diarrhea (67%), nausea (37%), and acneiform dermatitis (33%). Three patients (10%) experienced treatment-related adverse events leading to treatment discontinuation. Best response was stable disease in 14 patients (n = 10 with MK-8353/selumetinib 150/75 mg). Conclusion: MK-8353/selumetinib 50/25 mg and 100/50 mg had acceptable safety and tolerability, whereas 150/75 mg was not tolerable. No responses were observed.

**Supplementary Information:**

The online version contains supplementary material available at 10.1007/s10637-022-01326-3.

## Introduction

Selumetinib, an oral selective mitogen-activated extracellular signal-regulated kinase (MEK) 1/2 inhibitor, is approved for the treatment of pediatric patients ≥ 2 years old with neurofibromatosis type 1 and symptomatic inoperable plexiform neurofibromas [1–3]. Although MEK inhibitors alone have shown promise in certain cancers, not all patients respond to these agents [4]. Selumetinib monotherapy had modest activity in patients with advanced cancers, with objective response rates of up to 15% [5]. Moreover, despite an initial response to MEK inhibitors, acquired resistance can often develop through different mechanisms including reactivation of the mitogen-activated protein kinase pathway and subsequent restoration of extracellular signal-regulated kinase [ERK] 1/2 signaling) [4, 6]. MK-8353, a selective, orally available, adenosine triphosphate−competitive, small molecule inhibitor of ERK1/2, inhibits the kinase activity of ERK1/2 and induces a conformational change in ERK1/2 that prevents its phosphorylation and activation by MEK [7, 8]. Antitumor activity of MK-8353 was demonstrated in various human cancer xenograft models [7]. In a phase 1 study, 20% (3/15) of patients with advanced solid tumors receiving MK-8353 300−400 mg twice daily experienced a partial response, and 400 mg twice daily was considered safe and well tolerated [7]. Evidence also indicates the potential for acquired resistance to ERK inhibitors, with preclinical data suggesting this might be overcome by MEK inhibition [9]. The potential benefit of combination therapy with ERK and MEK inhibitors is therefore of interest. In preclinical models, investigational ERK inhibitors (AZ6197 or AZD0364) plus selumetinib demonstrated greater antitumor activity versus each agent alone [10, 11]. We conducted a phase 1b study (NCT03745989) to evaluate safety and tolerability of MK-8353 plus selumetinib in patients with advanced solid tumors.

## Methods

### Patients

Eligible patients were ≥ 18 years old with a histologically/cytologically documented, locally advanced or metastatic solid tumor; had received or been intolerant to all treatments known to confer clinical benefit; had ≥ 1 measurable lesion by Response Evaluation Criteria in Solid Tumors (RECIST) v1.1; had Eastern Cooperative Oncology Group (ECOG) performance status of 0−1; and had adequate organ function.

Key exclusion criteria included clinically active central nervous system metastases and/or carcinomatous meningitis; thromboembolic or cerebrovascular events within ≤ 6 months; neuromuscular disorders associated with elevated creatine kinase; and certain ophthalmologic findings (intraocular pressure > 21 mmHg or uncontrolled glaucoma; current or history of central serous retinopathy or retinal vein occlusion; or retinal degenerative disease). Previous treatment with a MEK, ERK, or BRAF inhibitor was also an exclusion criterion.

### Study design and treatment

This phase 1b, international, open-label, dose-escalation study used a modified toxicity probability interval (mTPI) design [12], with a target dose-limiting toxicity (DLT) rate of ~30% applied to identify the maximum tolerated dose (MTD) for combination therapy. Up to 5 dose levels for each drug were planned to be evaluated in combination: MK-8353 50, 100, 150, 200, and 250 mg and selumetinib 25, 50, 75, 100, and 125 mg. The following MK8353/selumetinib dose combinations were investigated in sequence: 50/25, 100/50, 150/75, 200/75, 200/100, 250/100, and 250/125 mg. Each drug was administered orally twice daily (morning and evening) in a repeating cycle of 4 days on/3 days off per week for each 3-week cycle to minimize toxicity relative to continuous dosing. Patients received treatment until radiographically documented disease progression per RECIST v1.1, unacceptable toxicity, intercurrent illness, investigator decision, or patient withdrawal.

Starting doses were based on pharmacokinetic (PK) data and the MTD of each agent when administered alone. Dose escalation and de-escalation decisions were based on the mTPI and were dependent upon the number of patients enrolled and number of DLTs at each dose level. **Online resource**, **Table S1** describes the definition for DLTs, and **Table S2** describes the dose-finding rules per mTPI design.

### Assessments and endpoints

The primary objective was to determine safety and tolerability and to establish preliminary recommended phase 2 doses for combination therapy. Associated endpoints were DLTs, adverse events (AEs), and study drug discontinuations because of AEs. The secondary objective was to evaluate PK. Exploratory objectives included preliminary evaluation of efficacy and biomarker analyses (phosphorylation of ERK [pERK], serum interleukin-8 [IL-8], ^18^F-fluoro-deoxy-glucose positron emission tomography [FDG-PET]).

The DLT window of observation was cycle 1. AEs were monitored from study treatment initiation through 30 days (90 days for serious AEs) following cessation of study treatment and graded using National Cancer Institute Common Terminology Criteria for Adverse Events v4.0. Ophthalmic examinations were completed at screening, on day 1 of cycle 2, and every 8 weeks thereafter.

PK concentrations of MK-8353 and selumetinib (and its metabolite, desmethyl selumetinib) were used to derive PK parameters. Serial plasma samples were collected for PK analysis on days 1 and 4 of cycle 1 before the morning dose; at 1, 2, 4, 6, and between 8−12 h after the morning dose. Additional samples were taken before the morning dose and 1 and 4 h after the morning dose on days 1 and 4 of cycle 2 and days 1 and 4 of cycle 5. PK parameters were determined using standard noncompartmental methods with Phoenix WinNonlin v8.1 (Certara, Princeton, NJ). Area under the plasma concentration−time curve (AUC) was calculated using the linear up/log down trapezoidal rule.

Tumor imaging was performed at baseline, every 9 weeks in year 1, and every 12 weeks thereafter. Samples for assessment of biomarkers (including IL-8) were collected predose on days 1 and 4 of cycle 1 and on day 4 of cycles 2 and 5; additional blood samples for pERK assessment were collected on day 1 of cycles 2 and 5. FDG-PET was done at baseline and on day 4 of cycle 2 as a pharmacodynamic biomarker reflective of the study drug–induced effects.

### Statistical analysis

Safety data were analyzed in the all-patients-as-treated (APaT) population, which included all patients who received ≥ 1 dose of study treatment. The DLT-evaluable population included patients in the APaT population who had ≥ 75% of the planned dose per cycle for both agents and were observed for safety for 21 days as well as those who had < 75% of the planned dose but experienced a DLT.

DLT rates across different dose combinations in each dose combination sequence were estimated using isotonic regression under the assumption of monotonicity between the DLT rates and dose levels for each drug; 80% CIs were provided based on the Bayesian posterior credible interval with a prior distribution of Beta (1, 1).

## Results

### Patients

The study was conducted between February 22, 2019, and March 19, 2021. Thirty patients were enrolled (n = 3, MK-8353 50 mg + selumetinib 25 mg; n = 12, MK-8353 100 mg + selumetinib 50 mg; n = 15, MK-8353 150 mg + selumetinib 75 mg). Median (range) age was 61.5 (26−78) years, 16 patients (53%) were men, 21 patients (70%) had a baseline ECOG performance status of 1, and 93% had received previous cancer therapy (Table 1).

**Table 1:** Patient Demographics and Baseline Characteristics (APaT Population)

Characteristic	MK-8353 50 mg + Selumetinib 25 mg n = 3	MK-8353 100 mg + Selumetinib 50 mg n = 12	MK-8353 150 mg + Selumetinib 75 mg n = 15	Total N = 30
Age, median (range), y	59.0 (48−64)	59.5 (26−73)	66.0 (27−78)	61.5 (26−78)
Sex				
Men	3 (100)	5 (42)	8 (53)	16 (53)
Women	0	7 (58)	7 (47)	14 (47)
ECOG performance status score				
0	1 (33)	3 (25)	5 (33)	9 (30)
1	2 (67)	9 (75)	10 (67)	21 (70)
Received previous cancer therapy	3 (100)	12 (100)	13 (87)	28 (93)
First line	1 (33)	2 (17)	1 (7)	4 (13)
Second line	1 (33)	3 (25)	5 (33)	9 (30)
Third line	1 (33)	6 (50)	2 (13)	9 (30)
Fourth line	0	0	3 (20)	3 (10)
≥ Fifth line	0	1 (8)	2 (13)	3 (10)
Missing	0	0	2 (13)	2 (7)

APaT, all patients as treated; ECOG, Eastern Cooperative Oncology Group

Data are presented as n (%) unless specified otherwise

All patients discontinued study treatment (n = 17, disease progression; n = 6, physician decision; n = 4, AE; n = 3, patient withdrawal). Median (range) time on therapy was 49 (3−246) days, and patients participated in a median (range) of 3 (1−11) treatment cycles.

### Dose-finding

Among 3 patients who received MK-8353 50 mg + selumetinib 25 mg (lowest dose), no DLTs were observed and the dose was escalated to MK-8353 100 mg + selumetinib 50 mg. Among 11 patients who received MK-8353 100 mg + selumetinib 50 mg, 1 patient experienced a grade 3 DLT of urticaria. As designated by the mTPI design, dose was escalated to MK-8353 150 mg + selumetinib 75 mg. Among 14 patients in the MK-8353 150-mg plus selumetinib 75-mg group, 7 patients (50% [80% CI, 34−66%]) experienced grade 2/3 DLTs (n = 2 each of blurred vision [grade 2], retinal detachment [grade 2], and vomiting [grade 3]; n = 1 each of diarrhea [grade 3], macular edema, nausea, and retinopathy [all grade 2]; Table 2). In accordance with dose-finding rules per mTPI design, the occurrence of DLTs in 7/14 patients in the MK-8353 150-mg plus selumetinib 75-mg group dictated to deescalate to the next lower dose; thus further dose escalation was not conducted beyond this dose level and the MTD dose identified was MK-8353 100 mg + selumetinib 50 mg. All DLTs, except for grade 2 macular edema and grade 3 vomiting (n = 1 each), were resolved at data cutoff.

**Table 2:** Dose-Limiting Toxicities (DLTs) in the DLT-Evaluable Population^a^

Treatment Group	Patients, N	DLT	Patients with DLTs, n	DLT by Maximum Grade^b^			
				Grade 1	Grade 2	Grade 3	
MK-8353 50 mg + selumetinib 25 mg	3	Any DLT	0				
MK-8353 100 mg + selumetinib 50 mg	11	Any DLT	1				
		Urticaria	1	0	0	1	
MK-8353 150 mg + selumetinib 75 mg	14	Any DLT	7				
		Diarrhea	1	0	0	1	
		Macular edema	1	0	1	0	
		Nausea	1	0	1	0	
		Retinal detachment	2	0	2	0	
		Retinopathy	1	0	1	0	
		Vision blurred	2	0	2	0	
		Vomiting	2	0	2	0	

^a^DLT-evaluable population consists of patients that received ≥1 dose of study medication and satisfy 1 of the following criteria: (a) were observed for safety for 21 days after the first dose of assigned treatment, or (b) experienced a DLT prior to 21 days after the first dose of assigned treatment

^b^Only the highest reported grade for a given DLT is counted for the individual patient. There were no grade 4/5 DLTs.

### Safety

Twenty-six patients (87%) experienced treatment-related AEs (all grade 1−3; Table 3). Grade 3 treatment-related AEs occurred in 9 patients (30%) overall; those occurring in > 1 patient were diarrhea and vomiting (n = 3 each). Three patients (10%) experienced treatment-related AEs leading to discontinuation of study treatment: 2 in the MK-8353 100-mg plus selumetinib 50-mg group (diarrhea, n = 1; urticaria, n = 1) and 1 in the MK-8353 150-mg plus selumetinib 75-mg group (retinal detachment); each of these treatment-related AEs subsequently resolved. Study treatment was interrupted because of treatment-related AEs in 13 patients (43%; see Table 3). One patient in the MK-8353 100-mg plus selumetinib 50-mg group died because of aspiration pneumonia, which was not treatment related.

**Table 3:** Summary of AEs (APaT Population)

	MK-8353 50 mg + Selumetinib 25 mg n = 3	MK-8353 100 mg + Selumetinib 50 mg n = 12	MK-8353 150 mg + Selumetinib 75 mg n = 15	Total N = 30
Any AE	3 (100)	12 (100)	15 (100)	30 (100)
Treatment-related AEs^a^	2 (67)	9 (75)	15 (100)	26 (87)
Grade 1	0	4 (33)	1 (7)	5 (17)
Grade 2	1 (33)	2 (17)	9 (60)	12 (40)
Grade 3	1 (33)	3 (25)	5 (33)	9 (30)
Grade 4/5	0	0	0	0
Serious	0	0	2 (13)^b^	2 (7)
Led to discontinuation of study treatment	0	2 (17)^c^	1 (7)^c^	3 (10)
Led to interruption of study treatment	1 (33)^d^	1 (8)^d^	11 (73)^d^	13 (43)
Treatment-related AEs occurring in ≥ 10% of patients				
Diarrhea	1 (33)	7 (58)	12 (80)	20 (67)
Grade 1	0	6 (50)	3 (20)	9 (30)
Grade 2	1 (33)	0	7 (47)	8 (27)
Grade 3	0	1 (8)	2 (13)	3 (10)
Nausea	0	4 (33)	7 (47)	11 (37)
Grade 1	0	1 (8)	3 (20)	4 (13)
Grade 2	0	3 (25)	4 (27)	7 (23)
Acneiform dermatitis	1 (33)	3 (25)	6 (40)	10 (33)
Grade 1	1 (33)	3 (25)	3 (20)	7 (23)
Grade 2	0	0	3 (20)	3 (10)
Vomiting	0	2 (17)	5 (33)	7 (23)
Grade 1	0	1 (8)	2 (13)	3 (10)
Grade 2	0	1 (8)	0	1 (3)
Grade 3	0	0	3 (20)	3 (10)
Fatigue	0	0	5 (33)	5 (17)
Grade 1	0	0	2 (13)	2 (7)
Grade 2	0	0	3 (20)	3 (10)
Blood creatine phosphokinase increase	0	0	4 (27)	4 (13)
Grade 1	0	0	4 (27)	4 (13)
Abdominal pain	0	1 (8)	2 (13)	3 (10)
Grade 1	0	0	2 (13)	2 (7)
Grade 2	0	1 (8)	0	1 (3)

AE, adverse event; APaT, all patients as treated

^a^Determined by the investigator to be related to study treatment. Patients could have experienced ≥ 1 treatment-related AE.

^b^Vomiting (n = 2), diarrhea (n = 1)

^c^MK-8353 100 mg + selumetinib 50 mg: diarrhea (n = 1), urticaria (n = 1); MK-8353 150 mg + selumetinib 75 mg: retinal detachment (n = 1)

^d^MK-8353 50 mg + selumetinib 25 mg: diarrhea (n = 1); MK-8353 100 mg + selumetinib 50 mg: mucosal inflammation (n = 1); MK-8353 150 mg + selumetinib 75 mg: diarrhea and vomiting (n = 4 each); alanine aminotransferase increase, blurred vision, nausea, and retinal detachment (n = 2 each); aspartate aminotransferase increase, acneiform dermatitis, bacterial enterocolitis, fatigue, hypomagnesemia, macular edema, myalgia, rectal tenesmus, and retinopathy (n = 1 each)

Data are presented as n (%)

In the 2 lower dose combination groups, no patient experienced treatment-related eye disorders. In the MK-8353 150-mg plus selumetinib 75-mg group, 8 patients (53%) experienced grade 1/2 treatment-related eye disorders (n = 2 each of blurred vision, retinal detachment, and visual impairment; n = 1 each of eye irritation, glaucoma, macular edema, periorbital edema, and retinopathy).

### Pharmacokinetics

**Fig. 1:**
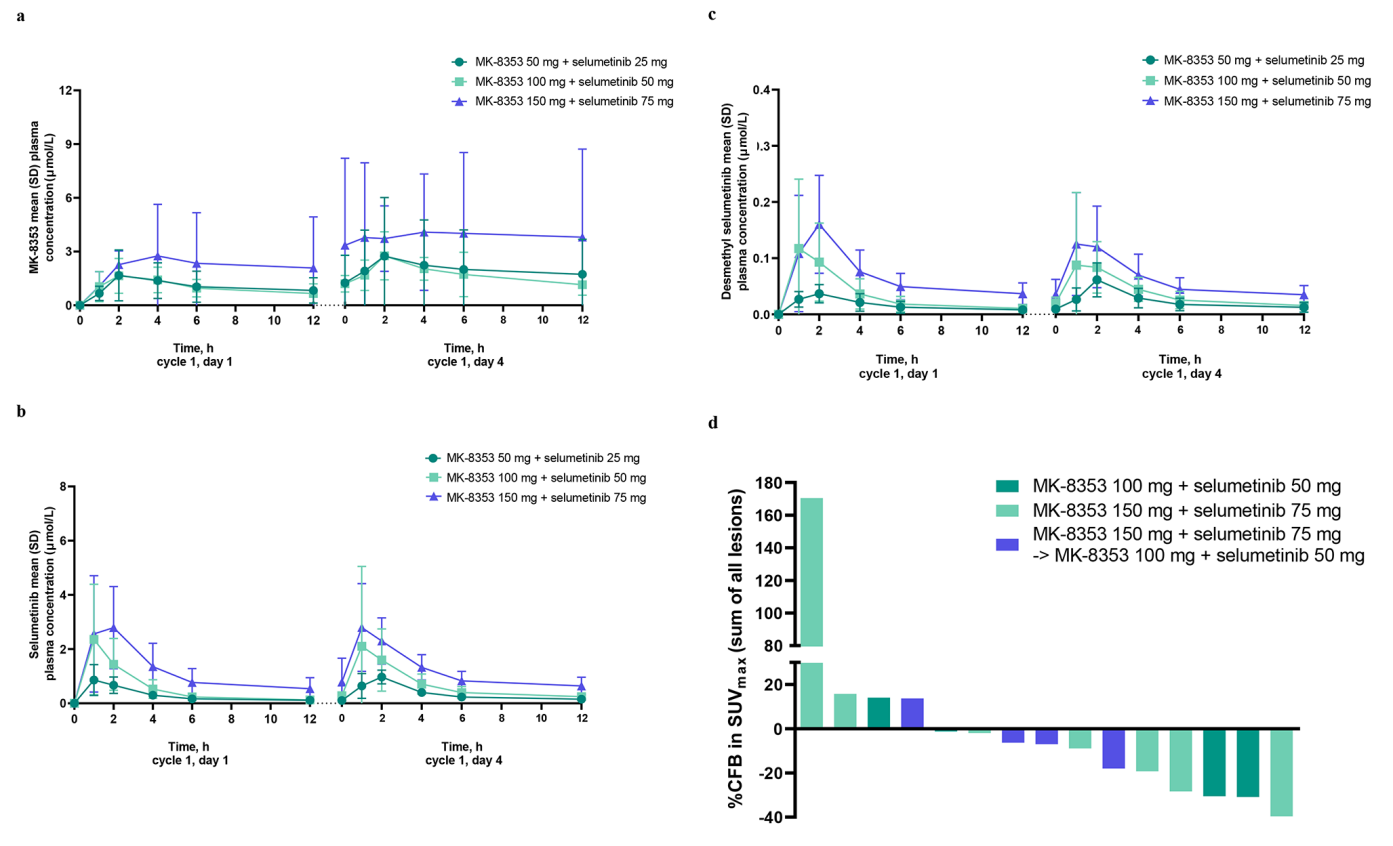
(a) Arithmetic mean plasma concentration versus time curves for MK-8353 following oral administration of the combination, (b) arithmetic mean plasma concentration versus time curves for selumetinib following oral administration of the combination, (c) arithmetic mean plasma concentration versus time curves for desmethyl selumetinib following oral administration of the combination, and (d) ^18^F-fluoro-deoxy-glucose positron emission tomography (FDG-PET) results in patients treated at a cycle 1, day 1 dose of MK-8353 100 mg + selumetinib 50 mg, MK-8353 150 mg + selumetinib 75 mg, and in patients where the dose reduction occurred before cycle 2, day 4 PET scans. CFB, change from baseline; SD, standard deviation; SUV, standardized uptake value

Plasma concentration versus time curves are shown in Fig. 1a–c. In cycle 1, geometric mean maximum plasma concentration (C_max)_ and area under the plasma concentration−time curve (AUC) of MK-8353, selumetinib, and desmethyl selumetinib increased with the dose level (Table 4). The observed median time of maximum plasma concentration ranged from 1.97−2.05 h for MK-8353 and 1.00−1.96 h for selumetinib and 1.00−1.96 h for desmethyl selumetinib. Geometric mean minimum plasma concentration (C_trough_) increased with increasing dose (0.695−1.47 µmol/L with MK-8353, 0.101−0.456 µmol/L with selumetinib, and 0.008–0.026 µmol/L with desmethyl selumetinib from lowest to highest dose). Minimal accumulation of up to approximately 2-fold was observed at steady state upon twice daily dosing for MK-8353, selumetinib, and desmethyl selumetinib.

**Table 4:** Plasma Pharmacokinetic Parameters (Cycle 1) of MK-8353 and Selumetinib Following Oral Administration of the Combination

	C_max_^a^, µmol/L	t_max_^b^, h	t_last_^b^, h	AUC_0−12_^a^, h∙µmol/L	AUC_0−last_^a^, h∙µmol/L
MK-8353					
50 mg					
Day 1 (n = 3)	1.24 (138.4)	2.05 (1.88−4.03)	8.02 (7.83−8.07)	14.3 (62.7)^c^	6.76 (117.8)
Day 4 (n = 3)	1.87 (149.4)	2.00 (1.87−5.88)	8.05 (7.97−8.08)	16.7 (365.7)^c^	10.7 (164.6)
100 mg					
Day 1 (n = 12)	1.65 (60.9)	2.00 (0.98−3.90)	8.00 (7.83−11.33)	9.66 (50.7)^d^	7.95 (63.1)
Day 4 (n = 10)^e^	2.53 (51.5)	1.97 (1.83−3.97)	8.00 (7.83−8.33)	19.0 (36.9)^d^	14.0 (46.8)
150 mg					
Day 1 (n = 15)	2.95 (62.3)	2.00 (1.85−7.95)	8.00 (7.83−8.37)	13.7 (32.8)^f^	13.9 (61.0)
Day 4 (n = 13)^g^	3.61 (80.9)	2.00 (0.00−8.00)^h^	8.00 (0.00−8.05)^h^	20.9 (60.6)^i^	23.4 (86.1)^j^
Selumetinib					
25 mg					
Day 1 (n = 3)	1.01 (29.7)	1.03 (0.92−1.88)	8.02 (7.83−8.07)	2.99 (27.2)	2.71 (29.0)
Day 4 (n = 3)	1.04 (13.3)	1.88 (0.87−2.00)	8.05 (7.97−8.08)	3.80 (24.6)	3.42 (21.7)
50 mg					
Day 1 (n = 12)	1.96 (91.1)	1.00 (0.88−2.00)	8.00 (7.83−11.33)	5.20 (63.2)	4.92 (65.7)
Day 4 (n = 10)^e^	1.44 (123.6)	1.96 (0.95−3.97)	8.00 (5.83−8.33)	6.28 (75.3)^k^	4.82 (80.5)
75 mg					
Day 1 (n = 13)	3.37 (57.1)	1.85 (0.83−4.00)	8.00 (7.83−8.37)	10.9 (49.1)^j^	10.2 (47.7)
Day 4 (n = 11)^g^	2.37 (107.5)	1.07 (0.00−5.92)^h^	8.00 (0.00−8.05)^h^	12.7 (31.5)^k^	11.0 (27.7)^f^
Desmethyl selumetinib					
25 mg					
Day 1 (n = 3)	0.0437 (10.5)	1.88 (0.92–2.05)	8.02 (7.83–8.07)	0.161 (52.4)	0.144 (47.7)
Day 4 (n = 3)	0.0563 (55.6)	1.88 (1.87–2.00)	8.05 (7.97–8.08)	0.230 (63.5)	0.202 (60.5)
50 mg					
Day 1 (n = 12)	0.0935 (124.8)	1.02 (0.88–2.08)	8.00 (3.92–11.33)	0.344 (73.5)^l^	0.261 (118.6)
Day 4 (n = 10)^e^	0.0724 (142.8)	1.96 (0.95–3.97)	8.00 (5.83–8.33)	0.386 (49.6)^k^	0.267 (102.1)
75 mg					
Day 1 (n = 13)	0.158 (68.9)	1.92 (0.83–4.00)	8.00 (7.83–8.37)	0.690 (56.3)^j^	0.547 (61.3)
Day 4 (n = 11)^g^	0.0977 (147.9)	1.07 (0.00–5.92)^h^	8.00 (0.00–8.05)^h^	0.681 (35.7)^k^	0.486 (87.7)^f^

AUC, area under the plasma concentration−time curve; AUC_0−12_, AUC from time 0 to 12 h; AUC_0−last_, AUC from time 0 to time of last quantifiable measure; C_max_, maximum plasma concentration; t_last_, time of last measurable plasma concentration; t_max_, time of maximum plasma concentration

^a^Geometric mean (percent geometric coefficient of variation)

^b^Median (range)

^c^n = 2

^d^n = 9

^e^For 1 patient, the actual sample collection time deviated substantially from the intended time; this patient was excluded prior to analysis

^f^n = 10

^g^One patient had only a predose sample available, which was below the limit of quantitation; this patient was excluded prior to analysis

^h^For MK-8353, t_max_ was observed at predose for 1 patient because C_max_ was observed at predose, and 1 patient had only a predose sample available with measureable concentration (t_max_ and t_last_ were therefore each observed at predose). For selumetinib and desmethyl selumetinib, 1 patient had only a predose sample available with measureable concentration (t_max_ and t_last_ were therefore each observed at predose)

^i^n = 7

^j^n = 12

^k^n = 8

^l^n = 11

### Exploratory analysis: antitumor activity

No patient achieved a complete or partial response. Fourteen patients experienced stable disease (n = 2, n = 2, and n = 10 at the 3 ascending dose levels, respectively).

### Exploratory analysis: biomarkers

In the subset of patients evaluated for biomarkers, maximal pERK inhibition in blood was observed at dose level 1 (~ 100% inhibition at 1 h postdose with return to near baseline levels after 3 days off treatment) and thus subsequent analyses were not deemed to provide additional information. Data regarding change in serum IL-8 were inconclusive owing to limited patient samples.

### Exploratory Analysis: FDG-PET Scans

Baseline and on-treatment FDG-PET scans were obtained from 15 patients (Fig. 1d). Greater than 30% decrease (the threshold for assessment of response in the PET Response Criteria in Solid Tumors [13]) from baseline in standardized uptake value was observed in 3/15 patients with no clear trend of dose-response relationship.

## Discussion

Combination therapy with the investigational ERK inhibitor MK-8353 and the MEK inhibitor selumetinib was tolerable at the 2 lower dose combinations evaluated (MK-8353 50 mg + selumetinib 25 mg and MK-8353 100 mg + selumetinib 50 mg). However, treatment with MK-8353 150 mg plus selumetinib 75 mg had unacceptable toxicity, with 50% of patients experiencing DLTs affecting the eyes and gastrointestinal system. Dose escalation was therefore stopped according to mTPI algorithm. The MTD (and therefore the recommended phase 2 dose) for the combination was MK-8353 100 mg plus selumetinib 50 mg. There were no responders at the dose levels assessed.

In a previous phase 1 study of patients with advanced solid tumors, no eye disorders occurred at the MTD of MK-8353 (400 mg twice daily), but visual impairment (n = 3), blurred vision (n = 2), and vitreous floaters (n = 1) occurred among 6 patients treated with the highest dose (800 mg twice daily) [7, 14]. As MEK inhibitors are known to cause ocular toxicity [15, 16], care was taken in our study to exclude patients with significant preexisting ophthalmologic conditions and to monitor for potential ocular AEs. Although the 2 lower dose combinations were not associated with any treatment-related eye disorders, the highest dose combination was associated with protocol-specified DLTs of grade 2 blurred vision (n = 2), retinal detachment (n = 2), retinopathy (n = 1), and macular edema (n = 1). The incidence of any treatment-related eye disorders in this arm (53%) was higher than rates reported in previous studies of selumetinib monotherapy (10−20%) and combination therapy with other agents including various chemotherapies and targeted therapies (up to 40%) [16], and may be indicative of additive toxicity from MK-8353 and selumetinib.

Gastrointestinal AEs were reported at the MTD of MK-8353 monotherapy (400 mg twice daily [n = 7]) in the aforementioned phase 1 study, including nausea (n = 4), diarrhea (n = 2), dyspepsia (n = 2), and vomiting (n = 2) [7, 14]. In a meta-analysis of 16 randomized controlled studies, patients who received MEK inhibitors had an increased risk of any-grade diarrhea or vomiting and grade 3/4 diarrhea versus controls [17]. The incidence of any-grade diarrhea and vomiting varied substantially, with ranges of 24–84% and 13–52%, respectively [17]. The incidences of treatment-related diarrhea and vomiting increased with increasing dose in our study but were within these ranges in all treatment arms.

The other DLT observed in our study was grade 3 urticaria (n = 1, MK-8353 100 mg plus selumetinib 50 mg). In the phase 1 study of MK-8353, maculopapular rash (n = 3), macular rash (n = 1), pruritus (n = 1), and urticaria (n = 1) occurred at the MTD (400 mg twice daily) [7, 14]. MEK inhibitors can increase the risk of dermatologic toxicities, with incidences in randomized controlled studies ranging from 22–76% for any-grade skin rash and 6–59% for any-grade acneiform dermatitis [18]. In our study, the incidences of treatment-related rash and maculopapular rash were low overall (3% and 7%, respectively), whereas the incidence of treatment-related acneiform dermatitis was greatest in the highest dose combination group evaluated (40%) but was within the range reported with MEK inhibitors in the literature [18].

Treatment-related cardiovascular AEs, which may be related to MEK inhibitors [19], were uncommon in our study. Treatment-related hypertension occurred in 7% of patients overall; no treatment-related cardiac disorders were reported.

On both day 1 and day 4 of cycle 1, the C_max_ of MK-8353 and selumetinib occurred within 2 h after dosing and appeared to increase proportionally over the doses evaluated. The PK profile of each compound in the combination was comparable to that observed for monotherapy [7, 20].

Previous clinical experience with the combination of an ERK inhibitor and MEK inhibitor is limited. In a phase 1 study, DLTs occurred in 1/3 patients with advanced solid tumors treated with the ERK inhibitor GDC-0994 200 mg plus the MEK inhibitor cobimetinib 40 mg once daily (grade 3 diarrhea) and 2/6 patients treated with GDC-0994 200 mg plus cobimetinib 80 mg once daily (grade 3 myocardial infarction, grade 3 rash and acneiform dermatitis) [21]. Cumulative toxicity could not be managed with supportive care, and the intolerability of the combination did not permit sustained dosing at levels that might have been therapeutic [21].

In our study, toxicity resulted in both study selumetinib and MK-8353 being administered at doses below their single-agent dose level which may have resulted in limited pathway inhibition [5, 7]. Such incomplete inhibition of the MEK and ERK signaling may also result in resistance via associated pathways (including the PI3K-AKT-mTOR pathway). If combinations of MEK and ERK inhibitors are to be evaluated in future studies, biomarker-based selection of patients whose tumors are oncogenically driven by MAPK pathway activation may be appropriate.

In summary, the combination of the ERK inhibitor MK-8353 plus the MEK inhibitor selumetinib did not demonstrate antitumor activity at tolerable doses. These findings are consistent with those from other studies that have evaluated combinations of MEK and ERK inhibitors in patients with advanced solid tumors.

## Electronic supplementary material

Below is the link to the electronic supplementary material.

Supplementary Material 1

## Declarations

### Data sharing statement

Merck Sharp & Dohme LLC, a subsidiary of Merck & Co., Inc., Rahway, NJ, USA (MSD) is committed to providing qualified scientific researchers access to anonymized data and clinical study reports from the company’s clinical trials for the purpose of conducting legitimate scientific research. MSD is also obligated to protect the rights and privacy of trial participants and, as such, has a procedure in place for evaluating and fulfilling requests for sharing company clinical trial data with qualified external scientific researchers. The MSD data sharing website (available at: http://engagezone.msd.com/ds_documentation.php) outlines the process and requirements for submitting a data request. Applications will be promptly assessed for completeness and policy compliance. Feasible requests will be reviewed by a committee of MSD subject matter experts to assess the scientific validity of the request and the qualifications of the requestors. In line with data privacy legislation, submitters of approved requests must enter into a standard data-sharing agreement with MSD before data access is granted. Data will be made available for request after product approval in the US and EU or after product development is discontinued. There are circumstances that may prevent MSD from sharing requested data, including country or region-specific regulations. If the request is declined, it will be communicated to the investigator. Access to genetic or exploratory biomarker data requires a detailed, hypothesis-driven statistical analysis plan that is collaboratively developed by the requestor and MSD subject matter experts; after approval of the statistical analysis plan and execution of a data-sharing agreement, MSD will either perform the proposed analyses and share the results with the requestor or will construct biomarker covariates and add them to a file with clinical data that is uploaded to an analysis portal so that the requestor can perform the proposed analyses.

## References

[1] Koselugo (selumetinib) (2021) Full Prescribing Information, AstraZeneca Pharmaceuticals LP, Wilmington, DE,

[2] Dombi E, Baldwin A, Marcus LJ, Fisher MJ, Weiss B, Kim A, Whitcomb P, Martin S, Aschbacher-Smith LE (2016). Activity of selumetinib in neurofibromatosis type 1-related plexiform neurofibromas. N Engl J Med.

[3] Gross AM, Wolters PL, Dombi E, Baldwin A, Whitcomb P, Fisher MJ, Weiss B, Kim A, Bornhorst M (2020). Selumetinib in children with inoperable plexiform neurofibromas. N Engl J Med.

[4] Kun E, Tsang YTM, Ng CW, Gershenson DM, Wong KK (2021). MEK inhibitor resistance mechanisms and recent developments in combination trials. Cancer Treat Rev.

[5] Ciombor KK, Bekaii-Saab T (2015). Selumetinib for the treatment of cancer. Expert Opin Investig Drugs.

[6] Guo YJ, Pan WW, Liu SB, Shen ZF, Xu Y, Hu LL (2020). ERK/MAPK signalling pathway and tumorigenesis. Exp Ther Med.

[7] Moschos SJ, Sullivan RJ, Hwu WJ, Ramanathan RK, Adjei AA, Fong PC, Shapira-Frommer R, Tawbi HA, Rubino J (2018). Development of MK-8353, an orally administered ERK1/2 inhibitor, in patients with advanced solid tumors. JCI Insight.

[8] Boga SB, Deng Y, Zhu L, Nan Y, Cooper AB, Shipps GW, Doll R, Shih NY, Zhu H (2018). MK-8353: discovery of an orally bioavailable dual mechanism ERK inhibitor for oncology. ACS Med Chem Lett.

[9] Jaiswal BS, Durinck S, Stawiski EW, Yin J, Wang W, Lin E, Moffat J, Martin SE, Modrusan Z (2018). ERK mutations and amplification confer resistance to ERK-inhibitor therapy. Clin Cancer Res.

[10] Decaudin D, El Botty R, Diallo B, Massonnet G, Fleury J, Naguez A, Raymondie C, Davies E, Smith A (2018). Selumetinib-based therapy in uveal melanoma patient-derived xenografts. Oncotarget.

[11] Flemington V, Davies EJ, Robinson D, Sandin LC, Delpuech O, Zhang P, Hanson L, Farrington P, Bell S (2021). AZD0364 is a potent and selective ERK1/2 inhibitor that enhances antitumor activity in KRAS-mutant tumor models when combined with the MEK inhibitor, selumetinib. Mol Cancer Ther.

[12] Ji Y, Li Y, Nebiyou Bekele B (2007). Dose-finding in phase I clinical trials based on toxicity probability intervals. Clin Trials.

[13] Wahl RL, Jacene H, Kasamon Y, Lodge MA (2009) From RECIST to PERCIST: evolving considerations for PET response criteria in solid tumors. J Nucl Med 50(1):122S-150S. 10.2967/jnumed.108.057307. 10.2967/jnumed.108.057307 PMC2755245 19403881

[14] National Institutes of Health. US National Library of Medicine (2021) A Study of the Safety, Tolerability, and Efficacy of MK-8353 in Participants With Advanced Solid Tumors (MK-8353-001). https://www.clinicaltrials.gov/ct2/show/study/NCT01358331?term=mk-8353&draw=2&rank=3. Accessed November 16, 2021

[15] Stjepanovic N, Velazquez-Martin JP, Bedard PL (2016). Ocular toxicities of MEK inhibitors and other targeted therapies. Ann Oncol.

[16] Mendez-Martinez S, Calvo P, Ruiz-Moreno O, Pardinas Baron N, Lecinena Bueno J, Gil Ruiz MDR, Pablo L (2019). Ocular adverse events associated with MEK inhibitors. Retina.

[17] Abdel-Rahman O, ElHalawani H, Ahmed H, Ellithy M (2015). Risk of selected gastrointestinal toxicities in cancer patients treated with MEK inhibitors: a comparative systematic review and meta-analysis. Expert Rev Gastroenterol Hepatol.

[18] Abdel-Rahman O, ElHalawani H, Ahmed H (2015). Risk of selected dermatological toxicities in cancer patients treated with MEK inhibitors: a comparative systematic review and meta-analysis. Future Oncol.

[19] Abdel-Rahman O, ElHalawani H, Ahmed H (2015). Risk of selected cardiovascular toxicities in patients with cancer treated with MEK inhibitors: a comparative systematic review and meta-analysis. J Glob Oncol.

[20] Banerji U, Camidge DR, Verheul HM, Agarwal R, Sarker D, Kaye SB, Desar IM, Timmer-Bonte JN, Eckhardt SG (2010). The first-in-human study of the hydrogen sulfate (Hyd-sulfate) capsule of the MEK1/2 inhibitor AZD6244 (ARRY-142886): a phase I open-label multicenter trial in patients with advanced cancer. Clin Cancer Res.

[21] Weekes C, Lockhart A, LoRusso P, Murray E, Park E, Tagen M, Singh J, Sarkar I, Mueller L (2020). A phase Ib study to evaluate the MEK inhibitor cobimetinib in combination with the ERK1/2 inhibitor GDC-0994 in patients with advanced solid tumors. Oncologist.

