# Supplementary material for: Results of an open-label phase 1b study of the ERK inhibitor MK-8353 plus the MEK inhibitor selumetinib in patients with advanced or metastatic solid tumors
Source: Invest New Drugs. 2023 Apr 11;41(3):380–90. doi: 10.1007/s10637-022-01326-3 (PMC10289957; doi:10.1007/s10637-022-01326-3)
Supplement: Supplementary file 1 — Supplementary Material 1 [file 10637_2022_1326_MOESM1_ESM.docx]

Results of an Open-Label Phase 1b Study of the ERK Inhibitor MK-8353 Plus the MEK Inhibitor Selumetinib in Patients With Advanced or Metastatic Solid Tumors

***Investigational New Drugs***

Anastasios Stathis, Anthony W. Tolcher, Judy S. Wang, Daniel J. Renouf, Lin-Chi Chen, Leah H. Suttner, Tomoko Freshwater, Andrea L. Webber, Tapan Nayak, Lillian L. Siu

Corresponding author: Anastasios Stathis, MD
Oncology Institute of Southern Switzerland
via A. Gallino 12
Bellinzona, 6500
Switzerland
Email: anastasios.stathis@eoc.ch

**Supplementary Appendix**

**Table S1. Definition of Dose-Limiting Toxicities (Cycle 1)**

| Grade 5 toxicity |
| --- |
| Nonhematologic toxicity |
| - Grade 4 toxicity (not laboratory) - Any grade ≥3 toxicity except grade 3 fatigue lasting ≤3 d; grade 3 diarrhea, nausea, or vomiting without use of antiemetics or antidiarrheals lasting <72 h; or grade 3 rash without use of corticosteroids or anti-inflammatory agents - Any grade 3 or 4 laboratory value if clinically significant medical intervention was required or if the abnormality led to hospitalization, persisted for ≥72 h, or was consistent with drug-induced liver injury |
| Hematologic toxicity |
| - Grade 4 toxicity lasting ≥7 d except thrombocytopenia (grade 4 thrombocytopenia of any duration or grade 3 thrombocytopenia associated with clinically significant bleeding) - Grade 3 or 4 febrile neutropenia |
| Liver toxicity |
| - Elevated AST or ALT ≥3 × ULN plus total bilirubin ≥2 × ULN and alkaline phosphatase <2 × ULN |
| Cardiac disorders |
| - Absolute decrease in left ventricular ejection fraction >10% compared with baseline and below the LLN of the institution - Grade ≥3 left ventricular systolic dysfunction - Any other grade ≥3 cardiac disorder |
| Vascular disorders |
| - Grade ≥3 hypertension requiring >1 drug or more intensive therapy - Grade 4 hypertension |
| Eye disorders |
| - Retinopathy or grade ≥2 retinal detachment confirmed by ophthalmic examination - Retinal vein disorder including retinal vein occlusion confirmed by ophthalmic examination - Grade ≥3 blurred vision, flashing lights, or floaters - Any grade 4 eye disorder confirmed by ophthalmic examination |
| Prolonged delay (>2 wk) in initiating cycle 2 because of treatment-related toxicity |
| Treatment-related toxicity causing discontinuation of study treatment in cycle 1 |
| Missing >25% of MK-8353 and/or selumetinib doses because of treatment-related adverse events in cycle 1 |

ALT, alanine aminotransferase; AST, aspartate aminotransferase; LLN, lower limit of normal; ULN, upper limit of normal.

**Table S2. Dose-finding rules per modified toxicity probability interval (mTPI) design**

|  | **Number of Participants Evaluable for DLT at Current Dose** | | | | | | | | | | | |
| --- | --- | --- | --- | --- | --- | --- | --- | --- | --- | --- | --- | --- |
| **Number of participants with at least 1 DLT** | **3** | **4** | **5** | **6** | **7** | **8** | **9** | **10** | **11** | **12** | **13** | **14** |
| **0** | E | E | E | E | E | E | E | E | E | E | E | E |
| **1** | S | S | S | E | E | E | E | E | E | E | E | E |
| **2** | D | S | S | S | S | S | S | S | E | E | E | E |
| **3** | DU | DU | D | S | S | S | S | S | S | S | S | S |
| **4** |  | DU | DU | DU | D | D | S | S | S | S | S | S |
| **5** |  |  | DU | DU | DU | DU | DU | D | S | S | S | S |
| **6** |  |  |  | DU | DU | DU | DU | DU | DU | D | S | S |
| **7** |  |  |  |  | DU | DU | DU | DU | DU | DU | DU | D |
| **8** |  |  |  |  |  | DU | DU | DU | DU | DU | DU | DU |
| **9** |  |  |  |  |  |  | DU | DU | DU | DU | DU | DU |
| **10** |  |  |  |  |  |  |  | DU | DU | DU | DU | DU |
| **11** |  |  |  |  |  |  |  |  | DU | DU | DU | DU |
| **12** |  |  |  |  |  |  |  |  |  | DU | DU | DU |
| **13** |  |  |  |  |  |  |  |  |  |  | DU | DU |
| **14** |  |  |  |  |  |  |  |  |  |  |  | DU |
| D = De-escalate to the next lower dose; DU = The current dose is unacceptably toxic; E = Escalate to the next higher dose; S = Stay at the current dose  Target toxicity rate = 30%. Flat noninformative prior Beta (1,1) is used as a prior and ε1=ε2=0.03. | | | | | | | | | | | | |
